# Digital Pulmonology Practice with Phonopulmography Leveraging Artificial Intelligence: Future Perspectives Using Dual Microwave Acoustic Sensing and Imaging

**DOI:** 10.3390/s23125514

**Published:** 2023-06-12

**Authors:** Arshia K. Sethi, Pratyusha Muddaloor, Priyanka Anvekar, Joshika Agarwal, Anmol Mohan, Mansunderbir Singh, Keerthy Gopalakrishnan, Ashima Yadav, Aakriti Adhikari, Devanshi Damani, Kanchan Kulkarni, Christopher A. Aakre, Alexander J. Ryu, Vivek N. Iyer, Shivaram P. Arunachalam

**Affiliations:** 1 GIH Artificial Intelligence Laboratory (GAIL), Division of Gastroenterology and Hepatology, Department of Medicine, Mayo Clinic, Rochester, MN 55905, USA; sethi.arshia@mayo.edu (A.K.S.); anmolmohanvan@gmail.com (A.M.); gopalakrishnan.keerthy@mayo.edu (K.G.); aakritiadhikari11@gmail.com (A.A.); 2 Department of Medicine, Mayo Clinic, Rochester, MN 55905, USAaakre.christopher@mayo.edu (C.A.A.); ryu.alexander@mayo.edu (A.J.R.); 3 Division of Pulmonary and Critical Care Medicine, Department of Medicine, Mayo Clinic, Rochester, MN 55905, USAiyer.vivek@mayo.edu (V.N.I.); 4 Department of Radiology, Mayo Clinic, Rochester, MN 55905, USA; 5 Microwave Engineering and Imaging Laboratory (MEIL), Division of Gastroenterology & Hepatology, Department of Medicine, Mayo Clinic, Rochester, MN 55905, USA; 6 Department of Cardiovascular Medicine, Mayo Clinic, Rochester, MN 55905, USA; yadav.ashima@mayo.edu (A.Y.); damani.devanshi@mayo.edu (D.D.); 7 Department of Internal Medicine, Texas Tech University Health Science Center, El Paso, TX 79995, USA; 8 INSERM, Centre de Recherche Cardio-Thoracique de Bordeaux, University of Bordeaux, U1045, F-33000 Bordeaux, France; kanchan.kulkarni@ihu-liryc.fr; 9 IHU Liryc, Heart Rhythm Disease Institute, Fondation Bordeaux Université, F-33600 Pessac, France

**Keywords:** respiratory disorders, lung sounds, AI, phonopulmogram, auscultation, electronic stethoscope, machine learning, deep learning

## Abstract

Respiratory disorders, being one of the leading causes of disability worldwide, account for constant evolution in management technologies, resulting in the incorporation of artificial intelligence (AI) in the recording and analysis of lung sounds to aid diagnosis in clinical pulmonology practice. Although lung sound auscultation is a common clinical practice, its use in diagnosis is limited due to its high variability and subjectivity. We review the origin of lung sounds, various auscultation and processing methods over the years and their clinical applications to understand the potential for a lung sound auscultation and analysis device. Respiratory sounds result from the intra-pulmonary collision of molecules contained in the air, leading to turbulent flow and subsequent sound production. These sounds have been recorded via an electronic stethoscope and analyzed using back-propagation neural networks, wavelet transform models, Gaussian mixture models and recently with machine learning and deep learning models with possible use in asthma, COVID-19, asbestosis and interstitial lung disease. The purpose of this review was to summarize lung sound physiology, recording technologies and diagnostics methods using AI for digital pulmonology practice. Future research and development in recording and analyzing respiratory sounds in real time could revolutionize clinical practice for both the patients and the healthcare personnel.

## 1. Introduction

Respiratory diseases are a major public health concern and a leading cause of mortality globally. According to the World Health Organization, respiratory disorders were among the top 10 global causes of death in 2019 and account for more than 8 million fatalities each year. The burden of these diseases is particularly high in low- and middle-income nations where access to healthcare is limited and air quality is suboptimal [1]. Chronic obstructive pulmonary disease (COPD) is the third-most common cause of death globally, causing 3.2 million deaths annually, while over 250 million individuals worldwide suffer from asthma [2,3,4]. Infections such as tuberculosis (TB) are also a significant contributor to the disease burden, with more than 10 million new cases and 1.4 million deaths annually [4,5,6]. Even in affluent nations, lung cancer remains one of the deadliest types of cancer, with a 5-year survival rate of just 10–20% [7]. The epidemiology of respiratory diseases highlights the need for increased efforts to prevent and manage these conditions.

Auscultation is a critical technique that is frequently used in conjunction with clinical and laboratory methods to diagnose respiratory illnesses. Auscultation is the process of using a stethoscope to listen to the chest to hear respiratory sounds and evaluate breathing patterns. This quick and easy technique offers crucial information for diagnosis [8]. In order to detect respiratory disorders, chest imaging and pulmonary function tests (PFTs) are also frequently performed. Chest X-rays (CXRs) or computed tomography (CT) visualize the chest, and PFTs assess lung capacity and function [9,10,11]. Bronchoscopy and biopsy entail inserting a scope into the airways in order to visually inspect the lung tissue up close and collect tissue samples for further examination. These techniques have helped to improve patient outcomes by supplementing correct diagnosis and treatment of respiratory disorders [12,13]. The field of pulmonary diagnostics is undergoing a significant transition as a result of the integration of artificial intelligence (AI) in healthcare, with AI algorithms assisting in the interpretation of imaging investigations, real-time analysis and clinical decision making [14].

Although successful, current techniques using traditional stethoscopes for identifying respiratory illnesses have some drawbacks, one of them being its subjective nature and dependence on the knowledge and expertise of the healthcare provider [15]. The intra-operator variability and subjectivity associated with auscultation leads to a lack in uniformity, which can make it challenging to evaluate and understand results over time or between various practitioners. The capacity of CXRs to identify several respiratory disorders, particularly in the early stages of disease, is also limited [16]. There are dangers associated with invasive diagnostic procedures such as bronchoscopy and biopsy, including infection and bleeding [17,18]. Additionally, patients in rural areas struggle to receive correct and timely diagnosis and treatment due to a lack of resources [19].

Lung sounds (LSs), or auscultation as they are commonly known, can be used to supplement diagnosis in several lung diseases, including pneumonia, bronchitis and asthma [8]. The variations in lung sounds can provide valuable information for both the diagnosis and treatment of respiratory diseases. Lung sounds can also be used to check on the success of respiratory disorder treatments [8]. Conventional stethoscopes, although non-invasive, quick and inexpensive, frequently provide weak sounds, making it challenging to recognize and identify some sounds, such as mild cardiac murmurs or pulmonary wheezes [20,21]. Since their normal frequency range is constrained, they frequently miss out on some high- or low-frequency sounds that can be crucial for diagnosis [22].

The need to increase the efficacy and accuracy of auscultation gave rise to the evolution of AI-based analysis. With previous definitions of pathological LSs in place, computer algorithms and programs have been developed to detect them automatically using the electrical recordings (done using electronic stethoscopes) of LSs known as phonopulmograms (PPGs). AI has the capacity for self-improvement as it learns from new data and cases and can be trained to perform better than traditional processing methods [23,24]. In recent years, AI algorithms have been used for the processing and recognition of LSs, among which the most frequently used algorithms include artificial neural networks, Gaussian mixture models and support vector machines [25]. Utilization of a digital stethoscope to record and store LSs of high quality and integration with AI enables the classification of LSs into normal or abnormal in real-time and serves as an essential screening step for physicians [21]. Asthma, COPD and pneumonia are among the respiratory illnesses that AI-based smart stethoscopes and machine learning algorithms are being utilized to identify; the stethoscopes can also analyze LSs and provide real-time feedback for more precise diagnosis [24,26]. AI-based systems can also be useful as a diagnostic tool to triage patients and identify those in need of serious care and referral to a specialist [24,26].

Over the years, there has been an evolution of technologies which have been able to pick up subtle clues to aid in diagnosis of respiratory sounds. While existing literature highlights the need for this technology, it fails to provide a clear understanding of the mechanism of LS production and its clinical usability. The purpose of this review was to study the physiology of LS origin, factors affecting its frequency, AI-assisted clinical applications and recording technologies in existing literature. Additionally, this review reflects on the prospects of using AI-assisted microwave-based dual sensing systems for PPG acoustics and lung tissue imaging and their impact on transforming pulmonology practice for improving patient care.

## 2. Physiology of Lung Sounds

LSs, believed to be the result of a structure–function continuum, are primarily produced by the airflow along the tracheobronchial tree. Ideally, the air should flow laminarly as it passes through the respiratory tract. However, laminar flow only occurs at small terminal components such as bronchioles. Usually when a large volume/tidal volume of air passes through the respiratory tract, it encounters branching and irregular-walled airways such as the trachea or bronchi, which causes a turbulent and haphazard airflow producing sound from the collision of air molecules with each other or with the airway walls [27]. Another mechanism for breath sound generation is the development of whirlpools between the 5th and the 13th generations of the bronchial tree [28]. The whirlpools or vortices are produced when air flows from a narrower circular circumferential opening into a wider one at the origin of these sounds inside the chest wall, ranging over a frequency of 50 Hz to 2500 Hz and possibly reaching up to 4000 Hz at the trachea [29]. However, as the sound traverses the lung parenchyma, pleura and chest wall, it becomes dampened over higher frequencies and the auscultated sounds over the chest wall are thus limited to a frequency range of 100–200 Hz [28].

**Figure 1:**
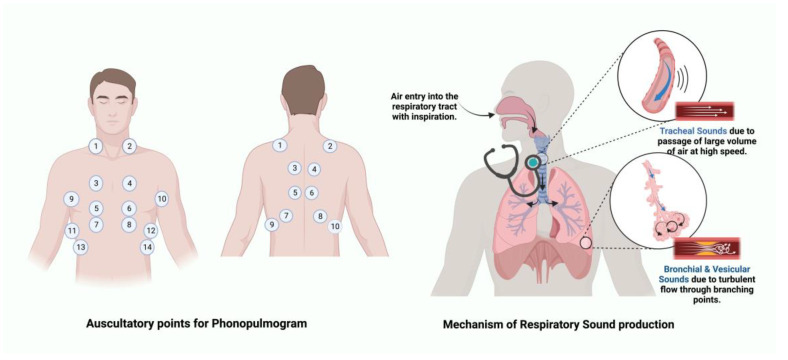
Physiological origin of lung sounds and primary sites of auscultation [30]. Numbers in the figure represent the points of auscultation.

LSs originate within the lungs, which differentiates them from the transmitted voice sounds originating from the larynx. LSs comprise normal breath sounds (Table 1) and adventitious/abnormal sounds (Table 2) as auscultated over the chest wall. The normal sounds can be further distinguished as normal tracheal sounds, normal or vesicular breath sounds or bronchial breath sounds, based on their characteristics and location of auscultation as shown in Figure 1. The various LSs are described in Table 1 and Table 2 [8].

## 3. Recording Technologies

Conventional stethoscopes, constrained by the subjectivity and expertise of the clinician, have found limited use in pulmonology practice [21,34]. Another drawback is their lack of use in telemedicine, remote care and care for COVID-19 patients because of personal protective equipment [35]. Stethoscope auscultation in busy clinic settings often results in poor signal transmission due to noise, tubular resonance effects and greater attenuation of higher-frequency sounds ranging from 50 Hz to 2500 Hz [34].

To overcome the shortcomings of a conventional pulmonary auscultation device, deep learning-based models through convolutional neural networks (CNNs) have been developed to enable electronic auscultation of LSs with digital stethoscopes for increased diagnostic accuracy and precision. In their comparative study of the effectiveness of doctor auscultation and machine learning-based analysis based on neural networks, Grzywalski et al. suggested that automatic analysis could increase efficiency [26]. A study revealed that AI algorithms were superior to physicians in detecting adventitious LSs [25].

Though machine learning has wide applications in analyzing LSs, the analysis is nonetheless constrained by the fact that it performs less accurately when the noises from the stethoscope itself and the surrounding environment are mixed into the recorded sounds or when two or more breathing sounds are present at the same time [21,36]. In addition, deep learning algorithms’ black box-type algorithmic aspect results in a certain lack of interpretability of the analyzed comprehensive information. Interpretability being a crucial component of the analysis, it also closely relates to technological challenges and data dependencies, hence the need for standardization and a clear definition [37]. Table 3 shows the various technological modalities and their shortcomings to gauge a better understanding.

### Implications and Limitations

Various AI models have been used in detecting and analyzing lung sounds [38,39,40,41,42,43,44,45,46,47,48,49,50,51,52,53,54,55,56,57,58,59,60,61,62,63,64,65,66,67,68,69,70,71,72,73,74,75,76,77,78,79,80,81]. These methods have been tested in and proposed to be used in a multitude of clinical settings. From classification of lung sounds using different models [40,41,42,44,45,46,48] to corelating lung sounds with degree of obstruction in asthmatics, AI shows promising capability. Along with its use in the diagnosis and management of various pulmonary disorders including pneumonia, COPD, asthma and IPF, its ability to filter cardiovascular sounds makes it superior to conventional stethoscopes [76]. Despite extensive research in the field, the lack of substantial sample sizes and the inability of current models to filter environmental noise have hindered AI’s development and use in everyday clinical practice [56]. Furthermore, since clinical decision making is feasible with interpretable AI, current reviews of AI models are mostly black box-type models without clarity regarding the exploitability of the features that relate to underlying pathophysiology in order to guide practice. AI researchers in this field should apply physiologically consistent signal processing and AI approaches with interpretable models that can augment physicians’ clinical decision making to diagnose and treat various lung diseases using phonopulmograms.

## 4. Clinical Applications of Lung Sounds

### 4.1. Infectious Respiratory Disorders

#### 4.1.1. Pulmonary Tuberculosis

In 2018, 10 million persons had incident TB and 1.5 people died of TB [82]. The sound properties of infected lungs differ from those of healthy lungs [83]. As a result, it is expected that infected lungs will exhibit adventitious LSs, which frequently signifies an abnormality in the lungs, such as obstruction in the airway passages or pulmonary disease. The lung damage brought on by active TB results in displaced lung tissue, which obstructs the airways and may cause wheezing. Crackles could be a sign of fibrosis brought on by the healing of the lungs [83]. In a study performed on healthy volunteers and patients with pulmonary TB, a large database of respiratory sounds was created and studied using multiple approaches, such as time domain, frequency domain and accidental wheezing and crackling analysis. The subjects in this study had their respiratory sounds recorded at 14 different sites on their posterior and anterior chest walls. The statistical overlap factor (SOF) was used to identify the most important signal characteristics in the temporal and frequency domains connected to the presence of TB. The auscultation recordings were then automatically classified into their respective groups for healthy or TB-origin using a neural network that was trained using these features. This study illustrates the potential of computer-aided auscultation in the detection and management of TB. Although the diagnostic accuracy of the neural network was 73% with automated noise filtering, more data training and potentially other signal processing techniques, the outcomes of future models can be enhanced. Such analyses will also enable follow-up with TB patients and gather more data as they receive treatment and recover to ascertain whether there is potential for complete recovery [84]. More in-depth research on electronic recording and digital analysis needs to be done on the peculiarities of respiratory sounds related to TB [84].

#### 4.1.2. Pneumonia

Every year, about 450 million people worldwide are affected by pneumonia, and delayed diagnosis results in about 4 million deaths. Respiratory sounds can be recorded with computerized stethoscopes and AI can be used to diagnose pneumonia with the gradient-boosting model, a machine learning model with an accuracy of 97%. This eliminates the need to perform CXRs, blood tests and pulse oximetry tests for diagnosing pneumonia, ensuring early diagnosis and management [80].

Signal processing and machine learning models are also used to classify normal, COPD and pneumonia patients with an accuracy of 99.7% according to a study conducted by Naqvi et al. [85]. Among children aged 1–59 months, hospitalized with WHO-defined clinical pneumonia without WHO danger signs (e.g., chest in-drawing, stridor, labored breathing, fast breathing), recorded LS analysis using machine learning and digital stethoscopes showed the presence of wheezing (without crackles) was correlated with lower odds of radiographic pneumonia and lower mortality as compared to children with normal recordings [86].

#### 4.1.3. COVID-19

CNN models have helped classify normal and abnormal lung sounds in COVID-19 patients and categorize them into normal, moderate, severe and critical cases with high accuracy and precision. Limitations existed in the study due to background noises interrupting lung sound analysis with CNN models. However, this method can provide clinicians with useful early prognostic information to facilitate pre-treatment risk stratification and guide medical staff to conduct more intensive surveillance and treatment of patients at high risk of severe illness to reduce mortality [35,79].

### 4.2. Non-Infectious Respiratory Disorders

#### 4.2.1. Restrictive Lung Disease

##### Interstitial Pulmonary Fibrosis

Patients with interstitial pulmonary fibrosis (IPF) frequently present with crackles similar to those of patients with pneumonia or congestive heart failure (CHF), leading to difficulty in diagnosis and potentially errors in management. Crackle pitch is one of the characteristics that notably differs between these diseases, which supports the widely held belief that IPF crackles are produced in smaller airways than those of CHF and pneumonia. Smither referred to the crackles of lung fibrosis brought on by asbestos as “characteristic in their sound and distribution,” and Wood and Gloyne described them as a major hallmark of this industrial disease as early as 1930 [87,88]. Using a 16-channel lung sound analyzer, 39 individuals with IPF, 95 with CHF and 123 with pneumonia were studied and machine learning techniques such as neural networks and support vector machines were used to assess crackle properties. With a sensitivity of 0.82, specificity of 0.88 and accuracy of 0.86, the IPF crackles could be distinguished from those in patients with pneumonia due to their distinctive features and with a sensitivity of 0.77, specificity of 0.85 and accuracy of 0.82, they were distinguished from those of CHF patients [61]. Fine crackles produced from a number of abnormally closed small airways increase in a lung with advanced fibrosis, which can be quantified by the machine learning-based analyzing algorithm. They were associated with the progression of lung fibrosis seen on high-resolution CT images in IPF patients, and the AI analysis had higher sensitivity than CXR findings of IPF [89]. Clinicians can make use of bedside computer analysis of crackles to diagnose IPF quickly and reduce medication errors [90]. In another study, patients with rheumatoid arthritis had their lung sounds recorded using an electronic stethoscope and analyzed using a Velcro sound detector (VECTOR) which showed a 93.2% sensitivity and hence proved to be a significant potential screening technique for rheumatoid arthritis patients with interstitial lung disease [90].

##### Asbestos-Related Lung Injury

Frequency distribution of lung sounds using computerized lung sound analyzer is significantly associated with interstitial lung fibrosis on high-resolution computed tomography (HRCT) scoring in patients with asbestos-related lung injury. The inspiratory crackles and high sound frequencies are associated with fibrotic changes to the lung while low sound frequencies were associated with emphysematous components of the asbestos-injured lung [91].

##### Pulmonary Edema

For many years, diagnosing pulmonary edema and tracking treatment response relied heavily on roentgenography and chest auscultation. Rales, which are now more commonly referred to as “crackles” in medical terminology, are still the primary auscultation feature used to diagnose pulmonary edema. Pulmonary edema can be cardiogenic and non-cardiogenic. One study described a multimodal sensing system that tracks changes in cardiopulmonary health by collecting data from bioimpedance spectroscopy, multi-channel lung sounds from four contact microphones, multi-frequency impedance pneumography, temperature and kinematics. The authors carried out a feasibility study on HF patients (*n* = 14) in clinical settings after initially validating the system on healthy people (*n* = 10). The ratio of resistance, from 5 kHz to 150 kHz (K), to respiratory timings (e.g., respiratory rate) were derived from three measurements conducted over the course of the hospitalization, and the researchers discovered an increase in K that was statistically significant (*p* < 0.05) from admission to discharge, as well as respiratory timings that were within physiologically reasonable limits. It was possible to identify Cheyne–Stokes breathing patterns and inspiratory crackles from patient recordings using integrated power (IP)-derived respiratory signals and lung sounds, respectively. This showed that the suggested system can record precise respiratory signals and lung sounds in a clinical scenario, as well as identify changes in pulmonary fluid status [92].

#### 4.2.2. Obstructive Lung Diseases

##### Chronic Obstructive Lung Disease

COPD causes the narrowing of air passages, making breathing difficult. The conventional methods of COPD diagnosis, via pulmonary function test, CXRs or AI-based analysis of CXRs or chest CT, are time-consuming, expensive and complex. Automated detection of LSs to diagnose COPD early can be timesaving for both the patient and the doctor. The physician can record and relay LSs to a pre-processing module, where it is augmented and passed to a convolutional neural network and classified into either COPD or non-COPD [72]. In addition, analyzing recordings from different auscultation points using multichannel lung sounds could help assess the whole lung rather than a specific region [93]. Machine learning models can also be used to predict acute exacerbation of COPD symptoms by telemonitoring computerized respiratory sounds, proving the significance of telehealth care systems for COPD management [72,94].

Computerized respiratory sounds are sensitive to short- and mid-term effects of pulmonary rehabilitation (PR) in patients with COPD. A study showed a decrease in inspiratory and expiratory median frequency of computerized respiratory sound related to improving the lung function of patients with COPD after PR in the band range of 100–300 Hz. Positive relationships between inspiratory median frequency and subjects’ symptoms (e.g., rest dyspnea, self-reported sputum) and health-related quality of life were found at the high-frequency band (300–600 Hz) [95].

##### Asthma

Lung wheezes can be detected by analyzing respiratory sounds’ frequency in asthma patients. High-pitched wheezing is associated with frequencies higher than 500 Hz. Frequency spectra in asthmatic patients can be categorized into three groups: asthma during an exacerbation, asthma in remission and normal state [41]. Several studies showed strong correlations between lung function parameter (FEV1) and median frequency of respiration sound power spectra computed from expiratory tracheal sounds, which can be established by computational techniques such as artificial neural networks [41,43]. A study by Islam et al. distinguished normal and asthmatic people using their posterior lung sound signals to reduce the inference of heart sounds, with the uniqueness of wheezing not being a necessary requirement for asthma detection [96].

Recently, studies have also identified asthma severity levels (mild, moderate and severe) by extracting integrated power features from respiratory sound signals, i.e., the energy of breath sounds in different sub-bands, which are not affected by airflow rate. In another study, the expiration/inspiration lung sound power ratio in a low-frequency band was used as a sign of airway obstruction and inflammation in bronchial asthma patients [66].

##### Cystic Fibrosis

The severity of lung disease can be monitored in cystic fibrosis (CF) patients using an artificial neural network with 89.05% average accuracy. Although conventional spirometry and a drop in FEV1% are commonly used tests to indicate the severity of lung disease in patients with CF, they require significant patient cooperation, especially in the pediatric population [66]. In a study by Karimizadeh et al., multichannel lung sounds were recorded from various regions of the lungs (large airways, upper airways and peripheral airways), expiration-to-inspiration lung sound power ratio features in different frequency bands (E/I F) were extracted and compared between the groups of different severity levels of lung disease using support vector machine, artificial neural network, decision tree and naïve Bayesian classifiers by the leave-one-sample-out method. Results showed that more severe lung disease occurred in the upper lobes compared to the lower lobes, hence discriminating between severity levels of CF lung disease [66].

##### Smoking and AI

Significant differences between digitally recorded respiratory sounds of healthy smokers and non-smokers have been noticed and can be used as the earliest indicator for detecting smoking-related respiratory diseases such as COPD, lung cancer, etc. [97].

#### 4.2.3. Lung Cancer

In one study, researchers showed how artificial neural networks were used to classify normal and crackle noises collected from 20 healthy subjects and 23 lung cancer patients, respectively. First, using a discrete wavelet transform (DWT) based on the Daubechies 7 (db7) and Haar mother wavelets, the sound data were divided into seven distinct frequency bands. Second, for five frequency bands (D3, D4, D5, D6 and D7), the detail coefficients’ mean, standard deviation and maximum PSD were computed as features. The ANN classifier took fifteen features as input. The classification results demonstrate that, when utilizing 15 nodes at the hidden layer, db7-based wavelets outperformed Haar wavelets with flawless 100% sensitivity, specificity and accuracy during the testing and validation phases. When utilizing 10 nodes at the hidden layer, Haar’s testing stage is the only one that demonstrated 100% sensitivity, specificity and accuracy [98]. However, we are looking for more literature regarding lung sounds in lung cancer to draw a definite conclusion.

## 5. Discussion

**Figure 2:**
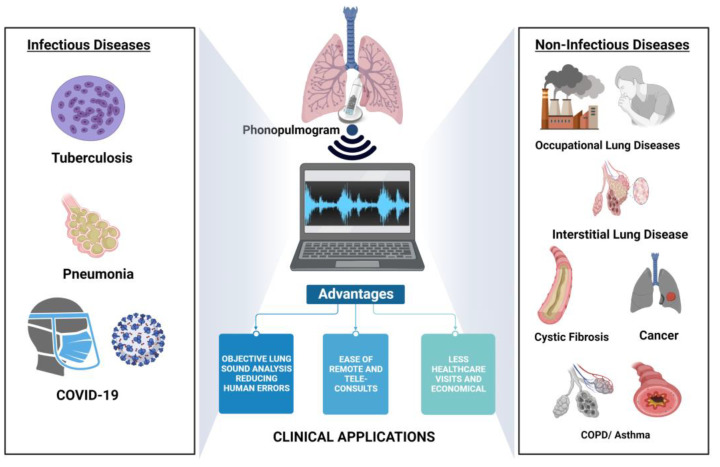
Pictorial representation of various AI-assisted clinical applications of phonopulmograms [30].

The pulmonary system is the site of the top 10 causes of mortality in 2019, with many fatal respiratory pathologies such as COPD, ILD and asthma and infectious diseases such as pneumonia, TB and most recently COVID-19. Rapid and easy screening has never been more essential [1,2,3,4,5,7]. While radiological investigations are an important confirmatory diagnostic method, initial pulmonary examinations by auscultation can help detect respiratory abnormalities [8,9,23]. Improvement and augmentation of the initial auscultation step could result in better screening of lung diseases as shown in Figure 2.

LSs, generated by the flow of air through the respiratory tract, can be altered by disruption of laminar flow [25]. Different types of LSs, both normal and abnormal, have been identified and studied which can help both guide diagnosis and monitor the progress of treatment. Utilizing these modulations of respiratory sounds, it has been possible to screen individuals with a respiratory pathology with the help of auscultation. However, the commonly used conventional stethoscopes are subject to inter-observer variability and produce weak sounds or sounds superimposed with background noise, posing a risk of missing out on certain sound frequencies essential for narrowing the diagnosis [21,22,23]. With the advent of digital stethoscopes, superior quality sound recording is possible and integration with artificial intelligence through neural networks, automated processing and analyses of sound recordings can offer a promising alternative [22,23,24]. Employing lung sound amplitudes, frequencies and timing as an input, an output of automatic breath sound identification is the goal of AI-integrated respiratory sound analysis [35].

Over the years, multiple technologies incorporating neural networks have been used to analyze LSs. In the 1990s and early 2000s, self-classifying networks and lung sound classification using back-propagation neural networks and wavelet transform methods showed high accuracy. Gaussian mixture models (GMM) were used to increase efficacy, with a hybrid of mel-frequency cepstral co-efficient and GMM showing higher reference recognition. This has been recently followed by using deep learning and machine learning-based classification of lung sounds and of pulmonary diseases ranging from pneumonia to COVID-19.

A phonopulmogram using machine learning methods could potentially transfigure respiratory clinical practice. There is a growing need for research with larger sample sizes and a standardized database of normal and pathological lung sounds, which could be used to diagnose patients quickly and efficiently. Such a system would also help patients who are in remote areas and unable to travel with the early diagnosis of disabling conditions such as interstitial lung disease and tuberculosis, thus significantly reducing both expenditure and strain on the healthcare system.

As illustrated in Figure 1, the 24 auscultatory sites could offer significant assistance in the simultaneous acquisition and analysis of phonopulmogram signals. Novel innovations in phonopulmogram acoustic sensor designs and implementation strategies are required to optimize effective acquisition across all auscultatory sites to obtain high-fidelity data for various lung diseases. AI-assisted sensor deployment will revolutionize the design and use of these sensors for specific lung diseases. Additionally, the data obtained from these acquisitions can further improve analysis of the phonopulmogram for effective detection, diagnosis and prognostication of various lung diseases as well as for treatment-monitoring applications. Standardized recording technologies and a dataset of various characteristic lung sounds will subsequently lead to the development of reliable AI-based models for automated lung sound analysis. These advancements will lead to an effective clinical decision support system that will impact digital pulmonology practice and reduce health care costs. The following section describes the author’s perspectives on the dual application of microwave systems for acoustic sensing of phonopulmograms as well as microwave imaging of the lung tissues for dielectric property measurement for a combined real-time digital assessment for improved patient care in pulmonology.

### AI-Assisted Microwave Based Dual Sensor System for Digital Pulmonology-Future Perspectives

Electrical impedance distribution in the human body is different as conductivity in each tissue is different. Conductivity also changes with pathology [99]. This principle has been used in electrical impedance tomography (EIT) imaging systems to diagnose various diseases. EIT is a new technology with clinical applications in specific lung pathology diagnosis, tumor detection and real time monitoring of lung volume changes [99]. Frerichs et al. conducted a study with EIT and reported that specific lung volumes related to spontaneous and mechanical ventilation could be separated, which helps optimize the ventilatory pattern for patients who are on artificial ventilation and therapy management for those patients [100]. Another study suggested that EIT can be used to identify imbalances in regional lung ventilation during mechanical ventilation [101]. EIT has also been used in diagnosing certain lung diseases. EIT reconstructs a cross-sectional image of lung conductivity, which correlates with regional ventilation. A study showed that EIT had 100% sensitivity in detecting pneumothoraces even with a small air volume in the pleural space [102]. The electrical properties of normal and diseased tissue in the human body are different. Bioimpedance studies help diagnose pathological tissues, including cancer [103]. Yang et al. conducted a multicenter study using electrical impedance analysis (EIA) as a diagnostic tool for pulmonary lesions. The study showed that EIA is an excellent diagnostic tool for lung cancers with high accuracy and can be adjunctively used with other diagnostic methods [103].

Similarly, microwave imaging (MWI) techniques are based on the dielectric properties of biological tissues. MWI uses electromagnetic waves at frequencies ranging from 0.5 GHz to 9.0 GHz to detect dielectric contrast that scatters from the tissue of the imaging domain [104]. Microwave (MW) technology can potentially help diagnose malignant tumors and other pathologies using the evaluation of complex permittivity of the tissue [105,106]. MW are safe diagnostic tools that generate images based on differences in dielectric properties. Recently, MWI has been gaining attention for diagnoses of various diseases such as breast cancer, bone tumors, stroke and lung cancer. Multiple studies have shown the difference in dielectric properties of ground glass opacities in lung lesions and the potential of MWI to detect these lesions [107].

Lin et al. conducted a study on detecting pneumonia in COVID-19 patients with MWI and showed promising results [107]. Khalesi et al. successfully experimented with Huygens principle-based MWI to see lung lesions in phantoms. The aim was to investigate elliptical, asymmetric and multilayer torsos. They suggested further research for a better MWI device that can be used in clinical trials for lung imaging [108]. Another study used a human torso to detect pulmonary edema and hemorrhage using MWI. They used a contrast source inversion method based on MWI and used the Cole–Cole model to determine the dielectric properties of human tissues. They simulated the scattered field via the method of moments [109]. The proposed technique shown in this study can potentially be used to locate and differentiate pulmonary edema and hemorrhage. Barbarinde et al. used a thorax phantom with simulated tissue dielectric properties and proposed that MWI can be potentially used for lung tumor detection. In this experiment, the microwave image was reconstructed using the delay-and-sum algorithm from the backscattered signals from the phantom [110]. Therefore, these studies show that MW-based techniques can be used as diagnostic methods for lung pathologies with improved antenna and hardware designs for clinical application.

There is an excellent development of electro-acoustic sensors based on electro-acoustic transduction in industrial, scientific and healthcare applications. Recently there have been tremendous advancements in acoustic biosensors, which are widely used to detect various diseases [111]. Microwave acoustic sensor applications have been used in power plants, aerospace and defense [112]. However, their use in healthcare is in its infancy; various research has been going on for the last few decades. Various acoustic techniques for pulmonary analysis have been discussed in the above sections. MW can also be used to detect acoustic parameters that can be used to develop diagnostic tools and biosensors with heart and lung sounds. Hui et al. developed the UHF microwave technique to retrieve heart sounds. They adapted previous near field coherent sensing (NCS) techniques [113]. This study demonstrated that MW NCS retrieved heart sounds similar to those retrieved by conventional stethoscope. With improvements to the antenna designs in the future, this technique could be used for clinical trials and as a sensor. The sound vibrations produced in the human body can be detected by MW technology. When the human body is subject to a low-intensity electromagnetic (EM) wave, the backscattered waves represent the object’s vibration along with amplitude modulation. This can be processed to retrieve helpful information on lung and heart functioning [114]. Microwave acoustic detection systems could be developed to detect signals created by the movement of the lungs, air and thoracic cavity. With further research and advancements in hardware design, microwave acoustic sensors for PPG sensing could be a promising diagnostic tool that can also be used for continuous patient monitoring.

**Figure 3:**
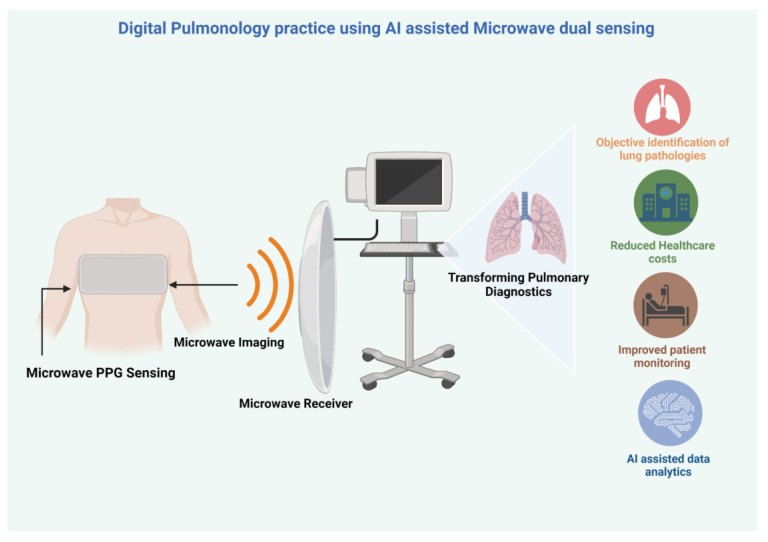
Pictorial representation of digital phonopulmography using AI-assisted dual microwave sensing systems [30].

It is evident that microwave-based sensors for a dual acoustic sensing for PPG and dielectric properties imaging are feasible with significant advancements in the AI-assisted microwave sensing and image reconstruction. Figure 3 depicts an implementation example of digital phonopulmography using dual microwave sensing systems and its potential impact. Novel microwave-based acoustic PPG sensors will open new avenues for technologies suitable for the accurate capture and recording of lung sounds. With an intelligent array of microwave antennas for dielectric property imaging, a combined dual sensing mechanism is feasible as a microwave belt that can provide simultaneous recording of LSs at the 24 auscultatory sites as well as microwave imaging of the target lung tissues. This will revolutionize practice by providing novel biomarkers for lung tissues when assessing various lung pathologies and its associated relations with lung sound analysis for providing novel digital insights in real time to improve clinical practice. Microwave telemetry may become inevitable with large microwave data capture and transmission to effectively operationalize digital pulmonology. AI-assisted methods are required in both PPG data and dielectric property data mining and interpretation as well as in the design of a computer-aided decision support system for the accurate diagnosis of various lung diseases. Digital phonopulmography using AI-assisted dual microwave sensing can positively impact pulmonology clinical practice operations as well as enhance patient care.

Future research is required on the design of novel AI-assisted microwave PPG acoustic sensors using enhanced metamaterial designs and frequency selective surfaces. Significant advancements are needed in microwave imaging hardware designs and robust dielectric properties reconstruction algorithms for accurate diagnosis of lung diseases. AI-assisted microwave telemetry system design is needed to provide noise-free PPG data transmission for reliable diagnosis. In this era of digital health, non-invasive diagnosis of lung diseases is warranted, employing novel AI-assisted microwave tools that can impact pulmonology practice and patient care. This review provides new insights and directions for practicing digital pulmonology using a dual sensing approach with microwave-based phonopulmography system.

## 6. Conclusions

Auscultation of lung sounds has been known to be a clinically useful technique for assisting with diagnosis of various lung diseases. Various studies have demonstrated the promising potential of digital lung sounds towards impacting pulmonology practice, though more research is warranted. While reasonable knowledge on the origin of normal lung sounds is well documented, further research is needed in better understanding the pathophysiology of abnormal lung sounds is needed to effectively translate acoustic features into clinical practice. The recording and analysis of lung sounds shows tremendous potential for the design and development of a patient friendly sensing device that can provide real-time analytics on the lung status. Microwave-based dual sensing approach for PPG sensing and microwave imaging for digital phonopulmography offers a huge opportunity to impact pulmonology practice as well as patient care. Technological advancements on the design of novel AI-assisted microwave acoustic and dielectric sensors with effective telemetry system designs will lead to an enhanced digital pulmonology practice in the future.

## Figures and Tables

**Table 1:** Normal Breath Sounds.

S. No.	Location	Mechanism of Production	Characteristics	Acoustics	Associated Pathological Conditions
1. Tracheal Sounds	Over trachea	Passage of large volume of air at high flow rate	Biphasic (inspiratory, expiratory) Harsh, high-pitched Expiratory phase > inspiratory phase	100–5000 Hz	
2. Bronchial Sounds	Manubrium (between 2nd and 3rd intercostal spaces)	Passage of air through progressively smaller airways	Hollow, high-pitched Biphasic (inspiratory, expiratory) Expiratory phase > inspiratory phase (I: E: 1:2) a/w Whispering pectoriloquy	Highly variable depending on site of auscultation and underlying pathology.	Consolidation Pleural effusion Alveolar collapse Mediastinal tumor
3. Vesicular Sounds	All over chest	Passage of air through bronchi and bronchioles	Soft, low-pitched Biphasic (inspiratory, expiratory) I: E: 2:1 Intensity of inspiratory phase> expiratory phase	Auscultation: 100–200 Hz Sensitive microphone: up to 800 Hz	

**Table 2:** Abnormal Lung sounds.

S. No.	Location	Mechanism of Production	Characteristics	Acoustics	Associated Pathological Conditions
1. Stridor [31]	Proximal/upper airway	Airway obstruction/narrowing	High-pitched Inspiratory (supraglottic narrowing/obstruction) Biphasic (glottis/subglottis/cervical trachea) Expiratory (thoracic trachea/bronchi)	>500 Hz	Adenoid hypertrophy, craniofacial abnormalities, choanal atresia, etc. (inspiratory) Laryngomalacia, vocal cord palsy, laryngeal mass, etc. (biphasic) Tracheal stenosis, foreign body, vascular compression, etc. (expiratory)
2. Wheeze [32]	Anterior/posterior chest wall	Airway narrowing (spasm/mass/mucus plugs/foreign body/parasite infestation)	High-pitched Shrill, coarse whistling/rustling Intensity on expiration > inspiration	100–5000 Hz	Asthma COPD Endobronchial mass Mucus plugging Foreign body
3. Rhonchus	Anterior/posterior chest wall	Passage of air through lower respiratory tract secretions	Low-pitched, squeaky Intensity on expiration > inspiration Character affected by coughing	~150 Hz	Pneumonia Chronic bronchitis Bronchiectasis
4. Coarse crackles [33]	Anterior/posterior chest wall	Passage and opening of airways clogged by secretions and fluids	Low-pitched Biphasic beginning at early inspiration	~350 Hz	Pulmonary edema Pneumonia Bronchiectasis
5. Fine crackles [33]	Anterior/posterior chest wall	Opening of collapsed terminal airways	High-pitched	~650 Hz	Interstitial lung diseases Congestive heart failure Pneumonia

**Table 3:** Summary of studies on technology applying AI in lung sound auscultation through phonopulmography to aid in disease detection.

Year, Author	Study	Technique	Results & Limitations
1989, Pasterkamp [38].	Assessing lung sound amplitudes, frequencies and timing using digital respirosonography.	Piezoelectric accelerometers → four-channel FM tape recorder → filtered and played through an analog-to-digital converter → IBM sensitive personal computer.	Sonograms of tracheal and vesicular sounds with sound intensities displayed on a color scale identified phases of respiration in normal and asthmatic patients.
1995, Forkheim et al. [39].	Testing of various neural networks to identify wheezes from different lung segments.	Raw data and Fourier transform data used to train and test back-propagation neural network (BPNN).	Fourier transform data provided a better classification rate than the raw signal using the BPNN with accuracy of 91%. Large training set is required to yield better results.
1997, Kahya et al [40].	Classification of lung sounds into obstructive, restrictive and healthy.	Autoregressive models applied to overlapping lung sounds were used to retrieve feature parameters using k-NN-voting and k-NN-multinominal classifiers. Leave-one-out method was used to classify.	The multinominal classifier showed higher performance in both expiration and combined inspiration and expiration cycle vs. the voting classifier.
1999, Rietveld [41].	Comparison of neural networks (NNs) and human examiners in classifying normal and asthmatic lung sounds.	Samples digitized and related to PEFR → Fourier spectrum was calculated from selected breath cycle → spectral vectors obtained were classified using NN.	Self-classifying networks were better (identified 96% of the spectrograms) at discriminating the classes of breath sounds than human examiners.
2000, Waitman et al. [42].	Representing and classifying breath sounds in an intensive setting.	Breath sounds represented by power spectral density → feature vectors → individual breath sounds → inspiratory and expiratory segments → number of inputs featured, hidden units and hidden layers calculated using BPNN.	The training tapes were better classified (91%) with a higher sensitivity (87%) and specificity (95%) vs. the ICU breath sounds (73%, 62% and 85%).
2000, Oud et al. [43].	Analysis of breath sounds produced by asthmatics and corelating them with degree of obstruction.	Air-coupled electret microphone attached to trachea → wireless tape-recorder → high-pass filtering → discrete Fourier transform (DFT) and Welch method → K-NN-based classifier.	Welch spectra are comparatively more convenient; 60–90% of the sound data classified according to their FEV1 value.
2002, Alsmadi et al. [44].	Digital signal processor (DSP) used for classification of lung sounds into healthy and pathological.	Microphone attached to the chest → breath sounds split into inspiration and expiration → segmented and modeled by an auto-regressive model of order 6 BY Levinson–Durbin algorithm → classified using k-NN and minimum distance classifier.	Encouraging results obtained for classifying sounds into two classes.
2003, Baydar et al. [45].	Automatic classification of respiratory sounds using signal coherence method.	Recorded breath sounds were amplified and digitized → signal coherence was calculated using the feature vectors.	Performance was unsatisfactory but could have promising application in wheeze analysis due to their sinusoidal structure.
2003, Bahoura et al. [46].	Classification of respiratory sounds using cepstral analysis.	Segmented sound characterized by fewer cepstral coefficients → classified using Vector Quantization (V) method.	Higher classification results vs. Autoregressive representation and the wavelet transform method of feature.
2004, Folland et al. [47].	Comparing constructive probabilistic neural network (CPNN) with multilayer perceptron (MLP) and radial-basis function network (RBFN) in classifying tracheal–bronchial breath sounds.	Data were presented as signal-estimation models of the tracheal–bronchial frequency spectra.	The classification by CPNN, MLP and RBFN was 97.8,77.8 and 96.2% accurate, respectively.
2004, Kandaswamy et al. [48].	Lung sound analysis using wavelet transform method.	Decomposed lung sounds into frequency sub-bands using wavelet transform → features extraction → classified using an artificial neural network (ANN)-based system.	Lung sounds classified into normal, wheeze, crackle, squawk, stridor or rhonchus.
2004, Gnitecki et al. [49].	Analysis of amplitude and patterns of lung sounds (LSs) in children before and after methacholine challenge test.	From root mean square (RMS) of LS and breath hold (BH) signals, signal-to-noise ratio (SNR) was determined, and 2 fractal-dimension (FD) algorithms based on signal variance and morphology were applied.	RMS-SNR and morphology-based FD values better classified bronchoconstriction with LSs.
2004, Bahoura et al. [50].	Classification of respiratory sounds into normal and wheeze using Gaussian mixture models (GMM).	Cepstral or wavelet transform used to characterize the sound signal divided into overlapping segments. This method compared with vector quantization (VQ) and multi-layer perceptron NN.	
2007, Chien et al. [51].	Classification of normal lung and wheezing sounds using cepstral analyses in Gaussian mixture models (GMM).	Lung sound recorded using electro-condenser microphone, amplified, filtered and analyzed using MFCC and Fourier transform-based model.	Accuracy of 90% at Gaussian mix 16 and increase in performance with longer length of time for training sound.
2008, Alsmadi et al. [52].	Using k-NN and minimum-distance classifiers to design an instrument to acquire, parametrize and classify LSs.	Sound signal from chest microphone and flow signal from pneumotachograph → feature extracted using LPC → classified based on 12 reference libraries	Clinical testing had a 96% accuracy.
2008, Lu et al. [53].	Automated crackle detection and classification.	Crackle separation, detection and classification using fractal dimension, wavelet packet filter (WPST) and GMM.	Separation using WPST 98%, detection sensitivity of 92.9% and a classification performance of 91.5%.
2009, Riella et al. [54].	Automatic wheeze detection in digitally recorded LSs.	Pre-processing of respiratory cycle → computing the spectrogram → stored as an array → multi-layer perceptron ANN.	84.82%, 92.86% accuracy for identification of wheeze in isolated and groups of respiratory cycle respectively.
2009, Bahoura et al. [55].	Comparing feature extraction by Fourier transform, linear predictive coding, wavelet transform and MFCC and classification using vector quantization, GMM and ANN.	Recorded sound split and extracted features used to train and test the model for classification.	Results achieved best by a combination of MFCC and GMM with *p* < 0.05 compared to other methods.
2009, Matsunaga et al. [56].	Segregation of normal and abnormal lung sounds based on maximum likelihood approach using hidden Markov models.	Two acoustic modeling methods were used: one for classifying abnormal sounds and the other for normal lung sounds.	Both models showed increase in recall rate for identifying abnormal and normal lung sounds. Noises hindered the improvement of recall rates.
2010, Mayorga et al. [57].	Evaluation and definition of lung sounds to assess relationship with respiratory diseases.	Electronic stethoscope to record lung sounds → analysis through GMM models to determine frequency of wheezing and crackles to predict disease state.	52.5% accuracy in cross-validation evaluation, 98.75% accuracy in reference recognition. This method could be used in <5-year-olds or to aid physicians with sensorial restrictions. Inability to process unwanted sounds and small sample size.
2010, Azarbarzin et al. [58].	Unsupervised snore classification algorithm of patients during their sleep.	LSs during sleep were recorded via polysomnography (PSG) by two tracheal and ambient microphones → detected with vertical box algorithm → K-means clustering algorithm to label as snore or no-snore.	Accuracy was 98.2% for tracheal recordings and 95.5% for ambient recordings. No requirement of prior training; robust and fast model.
2010, Flietstra et al. [59].	Automated analysis of crackles in interstitial pulmonary fibrosis (IPF) and ability to differentiate from crackles due to congestive heart failure (CHF) and pneumonia (PN).	Lung sounds from patients with IPF, CHF and PN were examined using a 16-channel lung sound analyzer and classified using neural networks and support vector machines (SVM)	IPF crackles were distinguished from PN crackles with a 0.82 sensitivity, 0.88 specificity, 0.86 accuracy. IPF crackles were separated from CHF crackles with 0.77 sensitivity, 0.85 specificity, 0.82 accuracy.
2011, Serbes et al. [60].	Novel method for crackle identification to aid in diagnosis of pulmonary disorders.	LSs with and without crackles → dual tree complex wavelet transforms (DTCWT) time-frequency (TF) and timescale analysis → feature subsets → SVMs	Usage of DTCWT enhances crackle detection ability of the model. Inability to use model in real time.
2011, Jin et al. [61].	Novel identification and extraction method of adventitious LSs based on instantaneous frequency (IF) analysis using temporal–spectral dominance-based features.	Electret condenser microphone to record LSs from healthy subjects and subjects with varying degrees of airway obstruction a TF decomposition method.	Accuracy of 92.4 ± 2.9%. Validity of results is required from more test subjects as well as pathological confirmation. Exploration of crackle LSs is required.
2011, Charleston-Villalobos et al. [62].	Assessment of parametric representation of LSs to classify them as normal or abnormal (ILD).	LSs → conventional power spectral density, eigenvalues of the covariance matrix and univariate autoregressive (UAR) and multivariate autoregressive models (MAR) → feature vectors→ supervised neural network.	The UAR model showed effectiveness with accuracy of 75% in healthy people and 93% in patients with ILD in LS parameterization.
2011, Yamashita et al. [63].	Distinction between healthy subjects and pulmonary emphysema patients based on LSs.	LSs → two-step classification process → hidden Markov models and bigram models → label acoustic segments as “confident abnormal respiration”.	Classification rate of 88.7% between diseased and healthy patients. Need for a refined threshold to finetune and improve performance.
2012, Xie et al. [64].	LS extraction using a multi-scale analysis system to aid in LS classification.	Healthy and pathological subjects with airway obstruction → multi-scale principal component analysis → enhance and extract signal → empirical classification.	Accuracy of 98.34%
2017, Gronnesby et al. [65].	Machine learning-based detection of crackles in lung sounds	Microphone with a recorder → reference database training sets with crackle and normal windows → preprocessing a classification and server implementation	5-dimensional vector and SVM with a radial-basis function kernel performed best with a precision of 0.86 and recall of 0.84.
2021, Karimizadeh et al. [66].	Multichannel LS analysis in determining severity of pulmonary disease in cystic fibrosis (CF) patients.	30-channel acquisition system → expiration-to-inspiration LS power ratio features calculated → support vector machine, ANN, decision tree and naïve Bayesian classifiers.	Upper and peripheral airways features were more effective in distinguishing between mild (91.1%) and moderate-to-severe (92.8%). The NN classifier had the best accuracy, of 89.05%.
2021, Chung et al. [67].	Artificial intelligence (AI)-based pneumonia diagnostic algorithm.	Loudness and energy ratio were used to represent the level of cough sounds and spectral variations.	90.0% sensitivity, 78.6% specificity and 84.9% accuracy.
2021, Nguyen et al. [68]	Transfer learning to tackle the mismatch of recording setup.	Pre-trained network used to build a multi-input CNN model.	F-score of 9.84% on the target domain.
2021, Ulukaya et al. [69].	Resonance-based decomposition to isolate crackles and wheezes.	Crackle and/or wheeze signals decomposed using tunable Q-factor wavelet transform and morphological component analysis	Significant superiority over its competitors in terms of crackle localization and signal reconstruction ability.
2021, Kim et al. [70].	Automated classification of breath sounds.	Deep-learning CNN to categorize LSs (normal, crackles, wheezes, rhonchi) → LS classification combining pretrained image feature extractor.	Accuracy of 85.7% and a mean AUC of 0.92 for classification of lung sounds.
2021, Ullah et al. [71].	LS classification.	LSs of varying duration → pre-processed segmented mel-frequency cepstral coefficients (MFCCs) and short-time Fourier transform (STFT) analysis → features used to train (70%) and validate (30%) models including ANN, SVM, K-nearest neighbor (KNN), decision tree (DT) and random forest (RF).	The best results were obtained with STFT + MFCC-ANN combination with an accuracy of 98.61%, 98% F1 score, 98% recall and 99% precision.
2021, Srivastava et al. [72].	CNN-based deep learning method for COPD detection.	Machine learning library features such as MFCC, mel-spectrogram, chroma and chroma CENS.	Classification accuracy score of 93%.
2021, Rani et al. [73].	Machine learning-based classification of pulmonary diseases from LSs.	LSs → Four machine-learning classifiers (SVM, KNN, naïve Bayes and ANN).	Low time complexity, robust and non-invasive.
2022, Nguyen et al. [74].	Classification of adventitious lung sounds and respiratory diseases.	Pre-trained ResNet model → vanilla finetuning, co-tuning, stochastic normalization and the combination of the three → data augmentation in both time domain and time frequency domain.	58.29 ± 0.24% and 64.74 ± 0.05% average score for the 4- and 2-class adventitious LS task and 92.72 ± 1.30% and 93.77 ± 1.41% average score for the 3- and 2-class respiratory disease classification tasks, respectively.
2022, Pancaldi et al. [75].	Automatic detection of pathological LSs in patients with COVID-19 pneumonia.	LSs of patients in the ER processed using software VECTOR, suitably devised for ILD.	Diagnostic accuracy of 75%.
2022, Wu et al. [76].	Overcoming subjectivity of conventional stethoscopes and filtering cardiopulmonary sounds.	An electronic stethoscope and an AI-based classifier recorded cardiopulmonary sounds which were then analyzed using fast FT.	Accuracy of 73.3%, sensitivity of 66.7%, specificity of 80% and F1 score of 71.5%.
2022, Neili et al. [77].	Evaluation and comparison of time frequency techniques such as spectrogram, scalogram, mel spectrogram and gammatone gram representations in lung sound classification.	LS signals obtained from the ICBHI 2017 respiratory sound database → converted into images of spectrogram, scalogram, mel spectrogram and gammatone gram TF → fed into VGG16, ResNet-50 and Alex Net deep learning architectures → network performances were analyzed.	Gammatone gram and scalogram TF images coupled with ResNet-50 achieved maximum classification accuracy.
2022, Vidhya et al. [78].	Diagnosis of pneumonia from lung sounds using gradient-boosting algorithm.	Electronic stethoscope → audacity software→ separates the required sound from unwanted noises.	Good identification properties with 97% accuracy.
2022, Dori et al. [79].	Full-spectrum auscultation device using machine learning analysis to detect COVID-19 pneumonia.	COVID, non-COVID patients, healthy LSs → full-spectrum stethoscope → machine learning classifier.	Sensitivity 97% and specificity 93%.
2022, Alqudah et al. [80].	Evaluation of different deep learning models in diagnosing respiratory pathologies.	Augmented datasets → three different deep learning models → generate four different sub-datasets.	Highest accuracy of CNN–LSTM model using non-augmentation was 99.6%, 99.8%, 82.4% and 99.4% for datasets 1, 2, 3 and 4.
2022, Kim et al. [21].	Diagnosing respiratory sounds using deep learning-based LS analysis algorithm		Overcoming the subjectivity of a conventional stethoscope.
2022, Kwon et [81]	Shifted δ-cepstral coefficients in lower-subspace (SDC-L) as a novel feature of lung sound classification	Performance of SDC-L evaluated with 3 machine learning techniques (SVM, k-NN, RF), two deep learning algorithms (MLP and CNN) and one hybrid deep learning algorithm combining CNN with long short-term memory (LSTM).	SVM, MLP and a hybrid deep learning algorithm (CNN plus LSTM) outperformed SDC-L, and the other classifiers achieved equivalent results with all features.

## Data Availability

The review was based on publicly available academic literature databases.
